# Progress in Pharmaceutical Sciences and Future Challenges

**DOI:** 10.3390/life14121636

**Published:** 2024-12-10

**Authors:** Ramón Cacabelos

**Affiliations:** International Center of Neuroscience and Genomic Medicine, EuroEspes Biomedical Research Center, 15165 Bergondo, Corunna, Spain; rcacabelos@euroespes.com

Over the past 25 years, pharmaceutical sciences have witnessed numerous groundbreaking discoveries that have transformed medical practice, improved patient outcomes, and expanded our understanding of disease mechanisms.

The most relevant discoveries in pharmacology from approximately 1999 to 2024 have been the following: (i) Targeted Cancer Therapies: the development of HER2 Inhibitors (e.g., Trastuzumab) revolutionized the treatment of HER2-positive breast cancer by specifically targeting the HER2 receptor, improving survival rates [1]. (ii) Immunotherapy and Checkpoint Inhibitors: the discovery of PD-1/PD-L1 Inhibitors (e.g., Pembrolizumab, Nivolumab) enabled the immune system to recognize and attack cancer cells, significantly improving outcomes in various cancers including melanoma, lung cancer, and more [2]. (iii) Pharmacogenomics and Personalized Medicine: understanding genetic variations in the CYP450 family has enabled personalized drug selection and dosing to minimize adverse effects and maximize efficacy [3]. (iv) Biologics and Monoclonal Antibodies: Rituximab was the first monoclonal antibody approved for cancer therapy, specifically targeting CD20 on B-cells, revolutionizing treatment for non-Hodgkin lymphoma and rheumatoid arthritis [4]. (v) CRISPR-Cas9 Gene Editing: the discovery of CRISPR-Cas9 Technology provided a precise tool for genome editing, opening new avenues for developing gene therapies for genetic disorders and advancing pharmacological research [5]. (vi) COVID-19 Therapeutics and Vaccines: the rapid development and deployment of mRNA vaccines (e.g., those developed by Pfizer–BioNTech and Moderna) have been pivotal in controlling the COVID-19 pandemic, showcasing a new platform for vaccine development [6]. (vii) Opioid Receptor Discoveries and Addiction Treatments: the discovery of buprenorphine as a partial opioid agonist provided an effective treatment for opioid use disorder with a lower risk of dependency and overdose compared to full agonists like methadone [7]. (viii) Advances in Drug Delivery Systems: nanoparticle-based drug delivery enhances targeted therapeutic delivery, improving drug solubility, stability, and bioavailability while reducing side effects [8]. (ix) Immunotherapy for Autoimmune Diseases: anti-TNF therapies (e.g., Infliximab, Adalimumab) have been used to successfully treat autoimmune conditions such as rheumatoid arthritis, Crohn’s disease, and psoriasis by targeting and inhibiting tumor necrosis factor (TNF) [9]. (x) Antiviral Therapies: direct-acting antivirals (DAAs) for Hepatitis C represent highly effective treatment regimens that can eliminate Hepatitis C infection in most patients, significantly reducing the incidence of liver cancer and cirrhosis [10]. (xi) Antibiotic Resistance and New Antibiotics: Linezolid, a novel oxazolidinone antibiotic, represented a new class of antibiotics effective against Gram-positive bacteria, including methicillin-resistant *Staphylococcus aureus* and vancomycin-resistant enterococci [11]. (xii) RNA Interference (RNAi) therapeutics: Patisiran, the first approved RNAi therapeutic, utilizes RNA interference to target and silence the production of a disease-causing protein in hereditary transthyretin amyloidosis, setting a milestone in gene-silencing therapies [12]. (xiii) Cannabinoid-Based Therapies: the FDA approved Epidiolex, a CBD-based drug, for treating seizures associated with rare forms of epilepsy (Dravet syndrome and Lennox–Gastaut syndrome). This opened the door for exploring further the application of cannabinoids as therapeutics [13]. (xiv) mTOR Inhibitors for Cancer and Autoimmune Diseases: Everolimus and Rapamycin analogs, inhibitors of the mammalian target of rapamycin (mTOR) pathway, have shown effectiveness in various cancers and as immunosuppressive agents in organ transplant recipients and people with autoimmune diseases [14]. (xv) Antisense Oligonucleotide Therapy: Nusinersen, an antisense oligonucleotide, was the first drug approved to treat Spinal Muscular Atrophy (SMA) by modulating SMN2 gene splicing, offering a groundbreaking therapy for a previously untreatable condition [15]. (xvi) Microbiome Research and Probiotic Therapies: the discovery of the gut microbiota’s critical role in health has led to the development of therapies that target the microbiome, with potential implications for treating metabolic disorders, autoimmune diseases, and mental health conditions [16]. (xvii) DPP-4 Inhibitors in Diabetes: DPP-4 Inhibitors (e.g., Sitagliptin) enhance the body’s natural ability to lower blood sugar by inhibiting dipeptidyl peptidase-4 (DPP-4), which breaks down incretin hormones [17]. (xviii) Monoclonal Antibodies for Alzheimer’s Disease: Aducanumab (Anti-Aβ Antibody) became the first monoclonal antibody targeting amyloid plaques in Alzheimer’s disease to be approved by the FDA, though its approval has sparked considerable debate regarding its efficacy [18]. (xix) PCSK9 Inhibitors for Cholesterol Management: Alirocumab and Evolocumab are monoclonal antibodies that inhibit PCSK9 and drastically reduce LDL cholesterol levels, offering an alternative for patients who cannot tolerate statins or who need further lipid-lowering therapies [19]. (xx) Gene Therapy for Inherited Retinal Dystrophy: Luxturna (Voretigene Neparvovec), the first gene therapy approved for the inherited retinal disease Leber congenital amaurosis, treats patients harboring a RPE65 gene mutation, restoring vision in individuals with the condition [20].

The three main health problems in developed countries are cardiovascular diseases (25–30%), cancer (20–25%), and nervous system disorders (10–15%). Significant progress is expected in the development of new treatment modalities for these three major groups of prevalent pathologies in the next decade. From a prospective point of view, key challenges in pharmacological sciences for the treatment of cardiovascular diseases, cancer, and central nervous system (CNS) disorders would be the following: (i) Cardiovascular diseases (CVDs) Challenges: (a) Precision Medicine and Genomics: The future of cardiovascular treatment lies in personalized medicine, which requires the better integration of genomics, proteomics, and metabolomics into clinical practice. The challenge remains in managing large datasets and understanding how these can guide tailored treatment strategies, particularly in hypertensive, atherosclerotic, and heart failure patients. (b) Targeting Inflammation and Immune Modulation: Chronic inflammation is a key driver of atherosclerosis and heart failure. While statins have anti-inflammatory effects, novel therapies targeting specific inflammatory pathways (e.g., IL-1 and IL-6 inhibitors) are still in development. The challenge is balancing efficacy and safety without causing immunosuppression. (c) Drug Resistance in Hypertension and Heart Failure: Many patients with hypertension or heart failure exhibit resistance to standard therapies like ACE inhibitors or ARBs. New drugs or alternative therapeutic pathways, including endothelin receptor antagonists or neprilysin inhibitors, are needed to overcome this challenge [21,22]. (ii) Cancer Challenges: (a) Overcoming Drug Resistance: Drug resistance, especially in metastatic cancers, is a major hurdle. Tumor heterogeneity, the tumor microenvironment, and genetic mutations (e.g., p53 mutations) contribute to resistance to chemotherapy, targeted therapies, and immunotherapies. New strategies are needed to overcome resistance, such as drug combinations or targeting resistance pathways. (b) Immunotherapy Enhancement: While checkpoint inhibitors (e.g., PD-1/PD-L1 and CTLA-4 inhibitors) have transformed cancer treatment, they are only effective for a subset of patients. The next challenge is improving response rates by overcoming resistance, increasing patient selection precision, and minimizing immune-related adverse events. (c) Targeting the Tumor Microenvironment: The tumor microenvironment plays a crucial role in cancer progression and resistance. Targeting stromal cells, angiogenesis, and immune cells within the tumor microenvironment presents both a challenge and an opportunity to develop more effective therapies [23,24]. (iii) CNS Disorder Challenges: (a) Blood–Brain Barrier (BBB) Penetration: A major obstacle in treating CNS diseases is the difficulty of delivering drugs across the BBB. Nanotechnology, focused ultrasound, and receptor-mediated transport systems are being explored to overcome this challenge, but practical and scalable solutions are still elusive. (b) Disease-Modifying Treatments for Neurodegenerative Diseases: Most treatments for Alzheimer’s, Parkinson’s, and other neurodegenerative diseases focus on symptomatic relief rather than halting disease progression. The discovery of new targets and biomarkers for early diagnosis and treatment is essential. For example, the role of tau, synuclein, and TDP-43 proteins in neurodegeneration remains underexplored. (c) Neuroinflammation as a Therapeutic Target: Neuroinflammation is increasingly recognized as a critical factor in the progression of diseases like Alzheimer’s, multiple sclerosis, and stroke. Targeting inflammatory pathways and microglial activation could offer new therapeutic opportunities, but understanding their precise role in CNS diseases remains a challenge [25,26,27,28].

Other neuropsychiatric disorders also deserve special therapeutic consideration, such as schizophrenia, depression, or epilepsy. The main challenge in schizophrenia is the limited effectiveness of current antipsychotics, particularly in treatment-resistant schizophrenia, and the inability to address negative and cognitive symptoms. For new mechanistic targets, studies are exploring glutamatergic and GABAergic pathways, as well as immune modulation, as potential new targets for treating schizophrenia. Emerging therapies, such as NMDA receptor modulators and anti-inflammatory agents, have shown promising preliminary results in clinical trials. In personalized medicine, pharmacogenetic research is advancing, with an increasing focus on identifying biomarkers that can predict drug responses, reducing side effects and improving outcomes [29,30].

The main challenges in depression are treatment-resistant depression (TRD) and the delayed onset of antidepressants. The following insights into depression have been gained: (i) Rapid-Acting Antidepressants: ketamine and esketamine continue to be at the forefront, with further research highlighting the potential of glutamate modulation in rapidly alleviating depressive symptoms; and (ii) Inflammation: A growing body of evidence suggests that inflammation may contribute to depression pathophysiology, especially in TRD. Anti-inflammatory agents and cytokine modulators are being investigated as novel treatments [31,32].

The major challenges in epilepsy are drug-resistant epilepsy (DRE) and a lack of neuroprotective therapies. Newer antiepileptic drugs (AEDs) are being developed with mechanisms targeting ion channels, neurotransmitters, and inflammation. Efforts are underway to incorporate gene therapy and neurostimulation techniques for DRE. Research is also focusing on developing neuroprotective treatments that could prevent neuronal damage associated with epilepsy. This could reduce comorbidities such as cognitive impairment [33,34].

The challenges in pharmacological treatments for neurodegenerative diseases, schizophrenia, depression, and epilepsy center around improving their efficacy, targeting the underlying disease mechanisms, addressing treatment resistance, and enhancing personalized treatment approaches. The integration of pharmacogenomics, novel targets, and rapid-acting agents is crucial for future drug development.
